# On-chip structure-switching aptamer-modified magnetic nanobeads for the continuous monitoring of interferon-gamma ex vivo

**DOI:** 10.1038/s41378-019-0074-1

**Published:** 2019-08-26

**Authors:** Guozhen Liu, Chaomin Cao, Shengnan Ni, Shilun Feng, Hui Wei

**Affiliations:** 1 Graduate School of Biomedical Engineering, ARC Centre of Excellence in Nanoscale Biophotonics (CNBP), Faculty of Engineering, The University of New South Wales, Sydney, NSW 2052 Australia; 2 Australian Centre for NanoMedicine, UNSW Sydney, Sydney, NSW 2052 Australia; 3 International Joint Research Center for Intelligent Biosensor Technology and Health, College of Chemistry, Central China Normal University, 430079 Wuhan, PR China; 4 School of Engineering, Faculty of Science and Engineering, Macquarie University, Sydney, NSW 2109 Australia

**Keywords:** Chemistry, Biosensors

## Abstract

Cytokines are cell signaling molecules that indicate the health status of the body. In this study, we developed a microfluidic device integrated with structure-switching aptamers capable of continuously tracking the concentration of the cytokine interferon gamma (IFN-γ) in cell culture medium and blood serum. First, a ferrocene (Fc)-labeled structure-switching signaling aptamer with a hairpin structure targeting IFN-γ was immobilized on magnetic nanobeads by the strongest noncovalent interactions between streptavidin and biotin. The aptamer-modified magnetic nanobeads were trapped on a customized microfluidic chip by a magnetic field to form the sensing interface. The binding of IFN-γ could trigger the hairpin structure of the aptamer to unfold, pushing Fc redox molecules away from the sensing interface and consequently switching off the electrochemical signal. The change in the redox current of Fc was quantitatively related to the concentration of IFN-γ in a linear range of 10–500 pg mL^−1^ and with the lowest detection limit of 6 pg mL^−1^. This microfluidic device was specific to IFN-*γ* in the presence of overabundant serum proteins and allowed the continuous monitoring of IFN-γ without adding exogenous reagents. It provided a universal point-of-care biosensing platform for the real-time detection of a spectrum of analytes.

## Sensors: High-sensitivity, real-time interferon gamma detector

Researchers have developed a device to continuously and precisely measure the concentration of the cytokine interferon gamma, a cellular signaling molecule which is an early indicator of many diseases, in real-time. Together with researchers in Asutralia and China, Dr. Guozhen Liu of the University of New South Wales engineered a DNA hairpin that recognized interferon gamma and changed the configuration upon binding, which was attached to magnetic nanobeads on a microfluidic chip. The resulting device can detect interferon gamma at concentrations of just 6 pg per mL. Detection occurs because the DNA hairpin includes a ferrocene molecule which moves away from the sensor when the hairpin unfolds in response to interferon gamma, changing the electrochemical signal. This device provides a sensitive point-of-care sensing platform for continuous screening of interferon gamma levels for understanding different health issues, and future work could reconfigure it to detect different target molecules.

## Graphical abstract

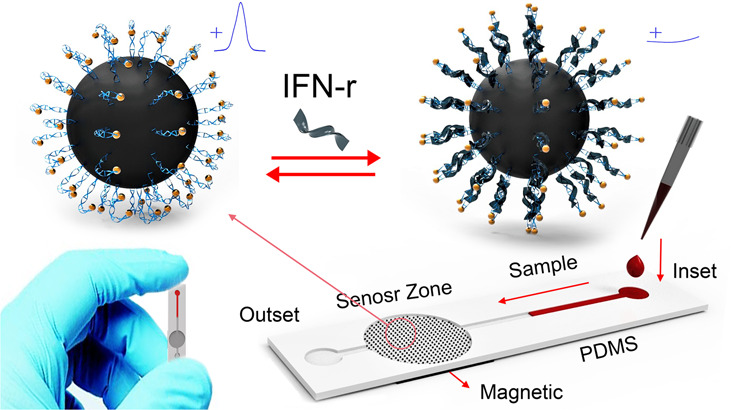

## Introduction

Cytokines are crucial cellular signaling molecules that are secreted by cells and are present in body fluids and tissues in the pM concentration range^1–3^. Elevated concentrations of cytokines normally accompany inflammation or disease^4^. Interferon gamma (IFN-γ) is a strong proinflammatory cytokine with direct antiviral activity, antiproliferative effects, and differentiation-inducing and immunoregulatory properties^5^. Consequently, elevated levels of IFN-γ can be an early indicator of multiple diseases such as tuberculosis^6^, HIV^7^, Crohn’s disease^8^, paratuberculosis^9^, and cancers^10^. The quantification of IFN-γ concentrations in serum and blood samples through various analytical techniques has been explored to further understand the immunological response during disease progression^7,11,12^. Current laboratory methods for detecting IFN-γ are based on a sandwich enzyme-linked immunosorbent assay (ELISA), enzyme-linked immunospot assay (ELISPOT), or reverse transcriptase polymerase chain reaction (RT-PCR)^13^. Though these techniques can detect IFN-γ at biologically relevant concentrations (10 pg mL^−1^ for tuberculosis^6^, 15 pg mL^−1^ for HIV^7^, and 10 pg mL^−1^ for Crohn’s disease^8^), they are arduous, expensive, and time consuming. Furthermore, these techniques, in most cases, require the transportation of samples and trained technicians to conduct the diagnostic tests in a laboratory setting. Thus, ELISA, ELISPOT, and PCR are generally not suitable for rapid, in-field biodetection^6,13^. These sensors usually perform only single point measurements and are incapable of continuously monitoring molecular analytes. The real-time monitoring of physiological species in body fluids has medical importance. For example, continuous monitoring of troponin I^14^, can predict a heart attack, and real-time tracking of cytokines can enable certain cancer patients to achieve the best treatment effect with minimal side effects^15^. To our knowledge, continuous real-time monitoring is currently possible only for a handful of molecular targets, such as glucose, lactose, and oxygen^16,17^. The few existing platforms for continuous molecular measurement rely on very specific chemistries (such as redox active or enzyme active) and are not generalizable for the monitoring of other analytes, such as proteins.

Biomolecular switches represent a particularly powerful approach to solve the problem of real-time molecular sensing in complex environments^18^. These biomolecules undergo binding-induced changes in conformation or oligomerization state to transduce chemical information into specific biochemical outputs. Structure-switching aptamer-based biosensors are a promising strategy for the versatile molecular monitoring of key analytes^18–20^. Recently, we summarized strategies based on structure-switching aptamer-based biosensors for real-time or near real-time monitoring of cytokines^21^. Additionally, the coupling and integration of a sensing system in a microfluidic device has successfully been applied in recent years for high-throughput real-time analysis and is able to process volumes of fluids on the order of nanoliters^22^. A simple microfluidic biosensor was developed to perform near real-time diagnostics of clinically relevant analytes such as cytokines and antibodies^23^. Revzin and coworkers designed a microfluidic device for the detection of local IFN-γ release from primary human leukocytes in real time with a sensitivity of less than 60 pM (1 ng mL^−1^)^24^. However, the sensitivity of this device is limited due to the low quantity of aptamers captured on the sensing interface.

Nanoparticles, especially magnetic nanobeads (MBs), have been steadily attracting interest in analytical science for the recognition of numerous analyte molecules with high sensitivity^25^. In addition to providing a large surface area to capture recognition molecules, the merits of MBs include their flexibility derived from functionalization by means of surface modification and specific binding, the simplicity of the washing and separation steps to exclude unspecific adsorptions, and the possibility of manipulating them inside microfluidic channels by utilizing high gradient magnetic fields. These advantages make them ideal candidates as the active component in miniaturized on-chip real-time biosensing systems. Several electrochemical applications based on MB technology for monitoring proteins^26,27^, toxins^28,29^, and nucleic acid hybridization^30,31^ have been introduced in the literature. To our knowledge, no report has been presented yet in the literature of the label-free amperometric detection of IFN-γ based on the fabrication of structure-switching signaling aptamers on microfluidic chips in combination with MBs assays.

**Fig. 1:**
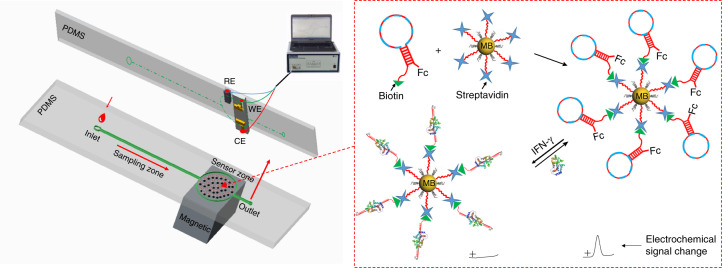
The schematic of the customized microfluidic chip integrated with magnetic nanobeads and structure-switching aptamers for the continuous monitoring of IFN-γ. The bottom part of the PDMS-based chip is the microfluidic pattern, including the sampling zone and sensor zone, and the top part is the PDMS-based chip with the sensing electrodes, including the working electrode (WE), reference electrode (RE), and count electrode (CE), on the silicon wafer. Both parts were bonded and combined by liquid PDMS

In this study, we developed a customized microfluidic chip that was integrated with MBs functionalized with the structure-switching signaling aptamers. This device was capable of continuously tracking the concentration of IFN-γ in cell culture medium and blood serum. First, a ferrocene (Fc)-labeled structure-switching signaling aptamer with a hairpin structure targeting IFN-γ was immobilized on MBs through interactions between streptavidin and biotin. The aptamer-modified MBs were trapped on a customized microfluidic chip by a magnetic field to form the electrochemical sensing interface. The binding of IFN-γ could trigger the hairpin structure of the aptamer to unfold, pushing Fc redox molecules away from the sensing interface and subsequently switching off the electrochemical signal (Fig. 1). This device was simple and easy to operate. It provided a universal point-of-care biosensing platform for the continuous screening of a spectrum of analytes ex vivo.

## Results

### Characterization of MB-aptamers

**Fig. 2:**
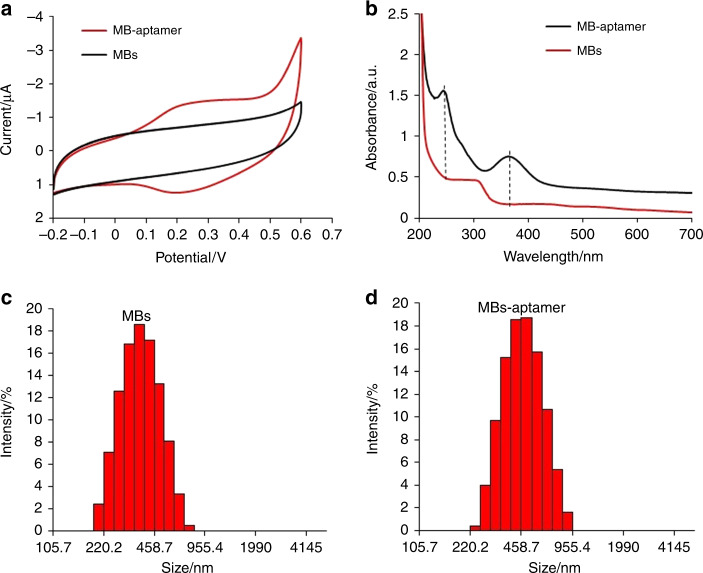
Electrochemical and DLS characterization of MBs and MB-aptamers. **a** Cyclic voltammetry measurements of MB in buffer C before and after reaction with 5 μM aptamer solution. **b** UV-Vis spectrum of MBs and MB-aptamer nanocomposites. **c** DLS distribution of MBs. **d** DLS distribution of MB-aptamer nanocomposites

The streptavidin of MBs tended to bind selectively to biotin labels on aptamers, mainly through strong noncovalent interactions^32^. The reaction between MBs and aptamers was optimized as shown in Fig. S2: the reaction of 30 μL of aptamer solution (5 μM) with 60 μL of MB solution (100 ng mL^−1^) demonstrated the highest current, suggesting that the maximum amount of aptamers was loaded on the surface of the MBs to form the MB-aptamer nanocomposites. To confirm the successful loading of aptamer on MBs, the prepared MB-aptamer nanocomposites were pumped down to a homemade microfluidic chip followed by electrochemical measurement in buffer C (Fig. 2a). According to the redox peak of Fc in Fig. 2a, the surface coverage of the Fc-labeled aptamer can be calculated to be 4.01 × 10^−11^ cm^2^ mol^−1^. Thus, the total number of aptamers (approximately 93,500) on the MBs in the sensor zone was calculated. As expected, the MBs did not show any Faradaic peaks between −0.2 and 0.6 V versus Ag/AgCl. For the MB-aptamer nanocomposites, redox peaks centered at 0.2 V were observed, which corresponded to the oxidation and reduction peaks of ferrocene, respectively, hence showing that aptamers were successfully attached to the surface^14^. These results were further confirmed by UV–vis spectra and dynamic light scattering (DLS) (Fig. 2b, c). An obvious adsorption peak at 260 nm, characteristic of DNA, was observed in the UV–vis spectrum of MB-aptamer but not in that of MBs^33^. In addition, the adsorption peak of magnetic beads was shifted from 300 to 360 nm upon aptamer conjugation, which is consistent with the literature^34^. The average diameter of the MBs increased from 300 to 400 nm after aptamer conjugation, according to the DLS data in Fig. 2c.

### Response of the microfluidic on-chip device to IFN-γ

**Fig. 3:**
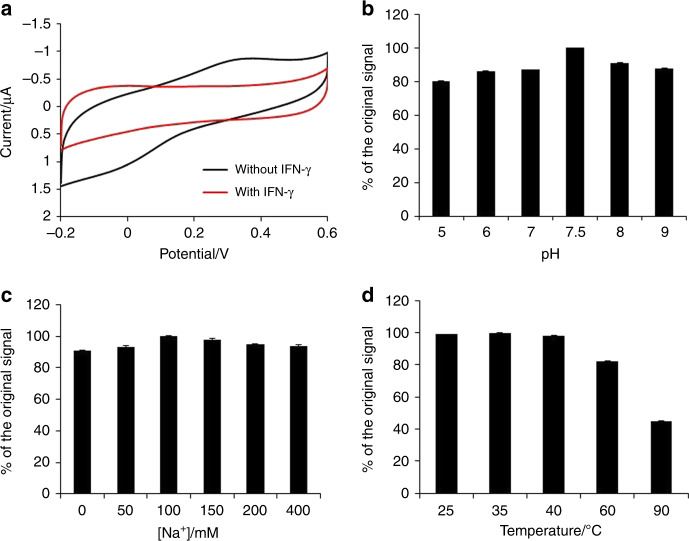
Response of the microfluidic on-chip device to IFN-γ under different conditions. **a** Cyclic voltammetry measurements of the on-chip sensing device in the absence and presence of 100 pg mL^−1^ IFN-γ. Effect of **b** pH, **c** salt concentration, and **d** temperature on the electrochemistry of the on-chip sensing device in the absence of IFN-γ

To ensure that all randomly organized MB-aptamer conjugates have the same chance to approach the electrode by forming a single layer of beads, the volume of MB-aptamer solution flowing through the microfluidic chamber was optimized. It was observed that 100 μL of MB-aptamer solution could form a thin layer of magnetic beads on the sensing zone, ensuring that all MB-aptamers were exposed to the analyte IFN-γ. Thus, the sensing device could provide the most reliable and sensitive electrochemical signal. Additionally, to ensure that there was sufficient interaction between the analyte IFN-γ and MB-aptamers on the microfluidic sensing device, the device flow rate was optimized. The flow rate of 16.7 μL/min provided the highest response to 100 pg mL^−1^ IFN-γ (Fig. S3). Figure 3 illustrates the electrochemical response of the MB-aptamer-modified microfluidic device after reaction with 100 pg mL^−1^ IFN-γ with an optimized flow rate of 16.7 μL/min. In the absence of IFN-γ, an obvious Faradic current was observed at 0.3 V, which is characteristic of ferrocene (Fig. 3a). However, the redox peaks disappeared after exposure to the analyte IFN-γ followed by washing with a copious amount of buffer B. The switching off of the electrochemical signal was due to the binding of IFN-γ to the loop part of the aptamers, leading to the opening of the stem parts and consequent pushing of ferrocene away from the electrode surface. This result suggests that the prepared on-chip sensor was usable for the detection of IFN-γ. According to the electrochemical response of the on-chip sensor surface to IFN-y (Fig. 3a), the average surface coverage of the MB-aptamer was calculated to be 1.45 × 10^−10^ ± 0.05 (*n* = 5) cm^2^ mol^−1^. The small standard deviation of 5% suggested that a similar amount of MB-aptamer could be repeatedly loaded onto the homemade microfluidic chip.

The sensor in this work is based on structure-switching aptamers, and the change in structural configuration is induced by the analyte, resulting in electrochemical signal switching. Thus, any factors that affect the folding of the aptamers on the MBs will have an effect on the sensor performance. The effects of pH, salt concentration, and temperature on the stability of the MB-aptamer sensing interface were investigated in the absence of IFN-γ^35–37^. We observed that the current of the sensing interface increased with pH from 5.0 to 7.5 after incubation with Tris-buffer solution for 5 min under different pH conditions (Fig. 3b). Under acidic conditions, the DNA was deprived of purines, leading to unfolding of the stem. Thus, a smaller number of ferrocene molecules were held away from the interfaces. However, when the pH reached 8, aptamers were denatured in these alkaline conditions, which neutralized the charge of the acids but also caused the hydrolysis of bases upon prolonged treatment. Thus, pH 7.5 was found to provide the optimized conditions for the combination between MB and aptamer. In addition, Fig. 3c shows that the aptasensor provided the maximum current when the salt concentration was 100 mM. Temperature also affects the release of ferrocene molecules (Fig. 3d). The release of ferrocene molecules was negligible before the reaction with IFN-γ at temperatures below 60 °C. Heating the MB-aptamer solution to 90 °C led to significant release of ferrocene molecules in the absence of IFN-γ. This effect is explained by DNA denaturation at this high temperature (90 °C), where a double strand separates into two single strands, resulting in decreased current. However, the MB-aptamer sensing interface performed well at a physiological temperature of 37 °C.

### Electrochemistry measurement of IFN-γ

**Fig. 4:**
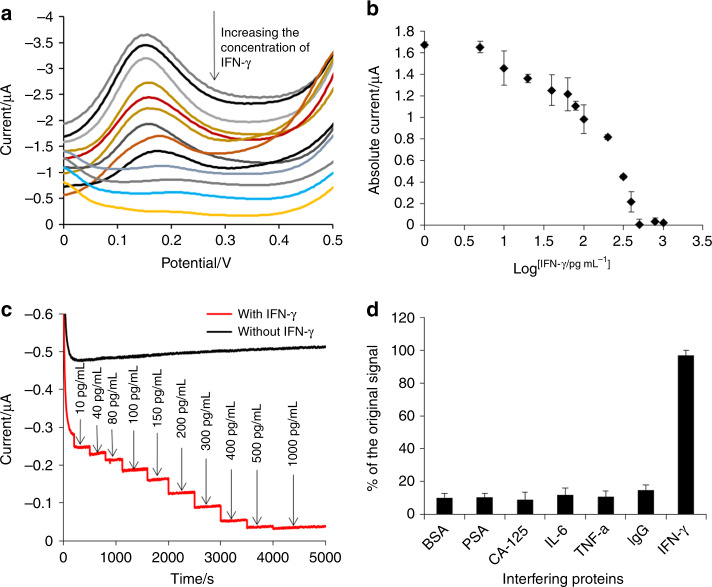
Performance of the microfluidic on-chip device for detection of IFN-γ. **a** SWV of the MB-aptamer microfluidic device after exposure to different concentrations of IFN-γ (0, 5, 10, 20, 40, 60, 80, 100, 200, 400, 500, 800, 1000 pg mL^−1^). **b** The relationship of the absolute peak current to the log concentration of IFN-γ. **c** The current record as a function of time for the MB-aptamer microfluidic device in buffer C solution at a constant potential of 0.2 V after the addition of IFN-γ at different concentrations. **d** Interference studies of MB-aptamer microfluidic device sensing interface responding to 500 pg mL^−1^ IFN-γ in the presence of nonspecific proteins (BSA, PSA, CA-125, IL-6, IgG, and TNF-α)

The performance of the on-chip device for detection of IFN-γ was studied under the optimized conditions (room temperature, pH 7.5, and salt concentration of 100 mM). Square wave voltammetry (SWV), a more sensitive electrochemical technique, can be used to quantitatively monitor IFN-γ. Figure 4a illustrates the SWV of the microfluidic chip device after incubation with IFN-γ at different concentrations. The Fc peak current decreased with the concentration of IFN-γ with a linear range of 10–500 pg mL^−1^, and the lowest detectable concentration of 10 pg mL^−1^ was obtained in buffer C. The detection limit was calculated to be 6 pg mL^−1^ based on three times the signal-to-noise with a confidence factor of 3. As determined from the midpoint (100 pg mL^−1^) of the calibration curve in Fig. 4b, the affinity constant between IFN-γ (molecular weight 16.9 kDa) and MB-aptamer is calculated to be approximately 2.1 × 10^10^ M^−1^. The typical values of the affinity constants *K*_a_ for antigen–antibody reactions are in the range of 10^8^–10^12^ M^−1^ ^38^, so this value indicates that the MB-aptamer probe has a very high affinity for IFN-γ. Additionally, a chronoamperometry experiment at a constant potential of 0.2 V, which was close to the oxidation potential of ferrocene, was carried out by adding different concentrations of IFN-γ (Fig. 4c). Upon the addition of IFN-γ, the monitored current of the MB-aptamer microfluidic device decreased accordingly, further suggesting that IFN-γ induced the configuration change of the aptamer leading to Fc far away from the interface. For the control experiment, PBS was added to the sensor zone, and no current switching was observed. This result further suggested that the signal switching of the sensing interface was caused by the binding of the analyte. The Fc signal tag was stable in the buffer solution. To study the specificity of aptamer sensors for IFN-γ, the electrochemical signal was monitored by incubation of the MB-aptamer sensor with the nonspecific proteins BSA (2 mg mL^−1^), PSA (1 mg mL^−1^), CA-125 (1 mg mL^−1^), IgG (1 mg mL^−1^), IL-6 (1 ng mL^−1^), and TNF-α (1 ng mL^−1^), respectively, without the presence of IFN-γ. Compared to the original signal, which was the response of the MB-aptamer sensor to 500 pg mL^−1^ IFN-γ in the absence of interfering proteins (Fig. 4d), no significant signal (less than 15%) was observed for the interfering proteins. This result suggests that this sensing interface has satisfactory specificity for IFN-γ. Meanwhile, the selectivity of the prepared microfluidic device sensing interface was studied by monitoring the electrochemical response of the MB-aptamer sensor to 500 pg mL^−1^ IFN-γ with the presence of the nonspecific proteins BSA (2 mg mL^−1^), PSA (1 mg mL^−1^), CA-125 (1 mg mL^−1^), IgG (1 mg mL^−1^), IL-6 (1 ng mL^−1^), and TNF-α (1 ng mL^−1^), respectively. The response of the sensing device to 500 pg mL^−1^ IFN-γ retained 95% of the original signal of 500 pg mL^−1^ IFN-γ in the absence of interfering proteins, suggesting a negligible degree of interference for these species tested in relation to the control test (<5%). To study the repeatability of the fabricated on-chip sensor surface, the standard deviation of the electrochemical response to the same concentration of 100 pg mL^−1^ IFN-γ was calculated to be 6.7%, indicating that the sensor results were repeatable.

### Stability and reversibility of the MB-aptamer sensors for detection of IFN-γ

**Fig. 5:**
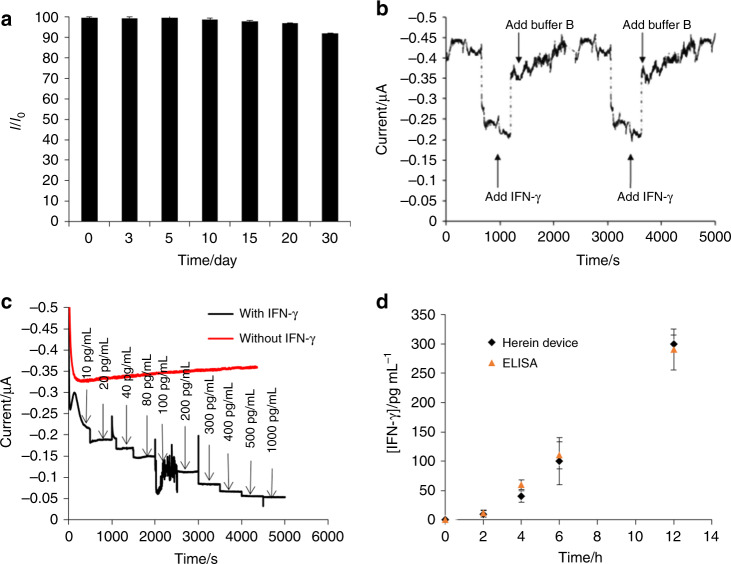
Stability and performance of the microfluidic on-chip device for detection of IFN-γ in real-time. **a** The stability of the MB-aptamer-based sensing interface. *I*_0_ is the signal obtained from the fresh sensing interface at day 0. **b** Chronoamperometry recording of the MB-aptamer-based sensing interface on the microfluidic device at a constant potential of 0.2 V after spiking in 100 pg mL^−1^ IFN-γ and buffer B, respectively. **c** Chronoamperometry recording of the MB-aptamer microfluidic device in cell culture medium of PBMCs at a constant potential of 0.2 V after spiking in IFN-γ at different concentrations and after spiking in PBS as a control. **d** Detection of IFN-γ in the supernatant of PBMCs after LPS stimulation for different periods of time using the MB-aptamer microfluidic device while comparing with ELISA

The stability of the MB-aptamer was investigated by recording time-dependent current changes in the MB-aptamer sensing interface, which was stored in PBS solution at room temperature over 30 days. *I*/*I*_0_ decreased slowly (Fig. 5a), and the signal (*I*) obtained at day 30 was 92 ± 2% of the signal (*I*_0_) from the fresh sensing interface at day 0, indicating that an ignorable amount (≤10%) of aptamer fell from the MB-aptamer sensing interface after storing in PBS for 30 days and that the noncovalent interactions between streptavidin and biotin were very stable. The binding between aptamers and proteins was a reversible process, which was studied on a magnetic glassy carbon electrode. The electrochemical signal before and after stirring the IFN-γ bound MBs in buffer solution for 1 h (Fig. S4) was recorded. This real-time detection capability of the on-chip sensing device was further investigated by recording chronoamperometry of the microfluidic device at a constant potential of 0.2 V after flowing through 100 pg mL^−1^ IFN-γ and buffer B (Fig. 5b). The electrochemistry of the chip device showed continuous signal-off and signal-on after the addition of the target analyte and the subsequent control buffer solution, further confirming the real-time detection capability. Additionally, the current retained over 95% of the signal of the freshly prepared MB-aptamer-based microfluidic device for detection of 100 pg mL^−1^ IFN-γ, suggesting that the on-chip sensing interface could be regenerated. The electrochemical signal switched off after IFN-γ binding. However, the signal recovered after the incubation of MBs in buffer B. This result suggests that upon binding to its target molecule, the probe underwent a reversible conformational rearrangement that modulated the redox current and generated an electrochemical signal^39^.

### Measurement of IFN-γ in cell culture media and while human blood serum

The MB-aptamer microfluidic device was used for the detection of IFN-γ secreted by live PBMCs (Fig. 5c). First, the chronoamperometry experiment at a constant potential of 0.2 V was carried out in the cell culture medium of PBMCs, to which IFN-γ at a concentration of 10–1000 pg mL^−1^ was added. It was observed that the current decreased continuously after adding IFN-γ until it reached a plateau when the concentration of IFN-γ was 500 pg mL^−1^. The control was carried out by recording the continuing current after spiking in PBS in the absence of IFN-γ. This result further suggested that the signal switching of the sensing interface was caused by the binding of the analyte. The Fc signal tag was stable in the cell culture medium. The supernatant of 1.0 × 10^6^ PBMCs was collected for IFN-γ analysis after LPS stimulation for 2, 4, 6, and 12 h by both the commercial ELISA kit and the microfluidic MB-aptamer device. As shown in Fig. 5d, the concentration of IFN-γ increased with the duration of the LPS treatment, and the highest content of IFN-γ was obtained after 12 h LPS stimulation. A similar trend was observed using an ELISA kit. The concentration values found by the two methods were statistically compared by means of Student’s *t*-test. The experimental *t* values (*t*_exp_, 0.30) were lower than the tabulated *t* value (2.11); therefore, no significant differences were found between the two methodologies at a significance level of 0.05. However, this herein sensing device has the capability of real-time cytokine sensing.

Finally, the developed microfluidic sensing device was applied to the determination of IFN-γ in human serum spiked at clinically relevant concentration levels. Regarding serum, the possible existence of matrix effects was initially evaluated by constructing a calibration plot in the sample (lyophilized serum from Sigma), which was spiked with IFN-γ. The equations obtained for the respective calibration graphs were *I* (µA) = 0.0023 C (pg mL^−1^) + 0.3198 for the detection of IFN-γ in serum. The comparison of the slope values with that (0.0026) calculated for the calibration plot constructed with standard solutions by applying Student’s *t*-test showed a *t*_exp_ value of 1.465, lower than the tabulated *t* of 2.426, therefore indicating that no statistically significant differences existed between the slope values for both types of calibration plots. Therefore, IFN-γ could be determined by interpolation of the current values measured for the serum samples into the calibration plots constructed with the standard solutions. Table 1 summarizes the results obtained by triplicate analysis of samples spiked at four different concentration levels: 50, 100, 150, and 200 pg mL^−1^ IFN-γ. Recoveries ranged between 98% and 101% with low relative standard deviations, indicating the reliability of the approach to determine low IFN-γ concentrations in serum following a simple working protocol. These results suggest that the microfluidic sensing device developed herein has the potential to determine IFN-γ at clinically relevant concentrations in biological fluids, such as serum, for point-of-care analysis.

**Table 1:** Recovery studies on human serum samples after spiking IFN-γ in different concentration

Serum samples	Added IFN-γ (pg mL^−1^)	Found IFN-γ (pg mL^−1^)	RSD (%)	Recovery (%)
1	50	49.9	3.18	99.7
2	100	101	1.15	101
3	150	147	2.00	98
4	200	199.3	2.30	99.6

## Discussion

Biosensing devices based on biomolecular recognition have attracted widespread attention for molecular analysis in food quality estimation, environmental monitoring, and the diagnosis of clinical and metabolic complications because of their simple instrumentation, low cost, and good portability. Especially with the aid of nanotechnology, the design of biosensors has undergone significant changes in the recent past. Recently, much efforts have been invested in the development of immunosensors for cytokine detection^40^. Cytokine immunosensing approaches provide powerful tools for the future of infectious disease diagnosis and drug screening^41^. However, the goal of reagentless, real-time biosensors for cytokine monitoring that can be deployed directly in complex samples remains largely unmet. A continuous and real-time biosensor for in vivo cytokine detection must be able to (1) selectively reject false signals that arise from interferants present in the complex environments found in vivo; (2) operate without requiring any exogenous reagents beyond those provided in situ by the organism; (3) operate continuously and not rely on batch process steps, such as separations, washing or incubation; and (4) provide a reversible response in concert with changing target concentrations. Unfortunately, although biomolecular recognition itself is enormously versatile, there is not yet any general method of adapting such recognition into sensors that support real-time, in vivo detection. The fundamental difficulty in the development of biosensors is the linkage of biomolecular binding to a specific, measurable output. For example, an antibody does not emit light or electrons upon binding to its target antigen. A common solution to this problem has been to attach the receptor to a surface and then measure a physical property, such as refractive index, mass, steric bulk, or charge, which changes when the receptor is occupied. However, these approaches suffer from a serious drawback: they cannot distinguish between the specific binding of target analytes and the nonspecific adsorption of contaminants. Thus, the existing challenge in the application of biosensors to in vivo cytokine monitoring is how to link specific cytokine secretion to measurable continuous output signals intracellularly, without invoking reagents or batch processing.

In this study, we take advantage of a microfluidic device, structure-switching aptamers, and MBs to successfully realize a sensitive and reliable point-of-care device for the real-time monitoring of cytokines in human blood serum. Chemosensing in nature relies on biomolecular switches or biomolecules that undergo binding-induced changes in conformation or oligomerization to transduce chemical information into specific biochemical outputs. The impressive performance (high specificity, affinity, and versatility of biomolecular recognition) of these natural “biosensors”, which support continuous, real-time detection in highly complex environments, has motivated decades of research on the development of sensors based on this effect^42^. Structure-switching molecules have demonstrated great potential in real-time biosensing^43^. When a suitably designed DNA aptamer binds to a specific analyte, it can change (switch) its structure, resulting in a measurable signal, and this effect can be used to design various sensing schemes to realize continuous monitoring^36^. Most biomaterials, such as antibodies, do not respond in any easily measurable way upon binding their target ligands. Additionally, due to their small size, lack of immunogenicity, and ease of chemical modification, structure-switching DNA aptamers are particularly promising as alternative molecular recognition elements to antibodies for molecular diagnosis with high sensitivity and specificity. The integration of switching molecules with microfluidic devices has significantly amplified the utility of structure-switching DNA aptamers for real-time detection, as demonstrated in this study. Furthermore, the sensitivity of the fabricated biosensing system has been improved with the aid of commercially available MBs, which provide a large surface area and make the washing steps easy and convenient.

## Conclusion

We present a customized disposable microfluidic chip modified with a MB-aptamer nanocomposite for the continuous detection of IFN-y in a wide range of concentrations with excellent precision. Our microfluidic electrochemical detector for in vitro continuous monitoring requires no exogenous reagents, operates at room temperature, and can be reconfigured to measure different target molecules by exchanging probes in a modular manner. In this paper, a DNA hairpin containing an IFN-γ binding aptamer was biotinylated, conjugated with a ferrocene (Fc) redox tag, and immobilized on magnetic beads by the strongest noncovalent interactions between streptavidin and biotin. The binding of IFN-γ caused the aptamer hairpin to unfold, pushing Fc redox molecules away from the electrode and decreasing electron-transfer efficiency. The change in redox current was quantified using SWV and was found to be highly sensitive to IFN-γ concentration. The limit of detection for the optimized biosensor was 6 pg mL^−1^ with a linear range of 10–500 pg mL^−1^. The sensitivity was approximately three orders higher than that in the literature (6.35 ng mL^−1^)^44^, which might be due to the MBs providing a large surface area. This microfluidic sensing device was specific to IFN-γ in the presence of overabundant serum proteins and allowed the continuous monitoring of IFN-γ without adding exogenous reagents. It provided a universal point-of-care sensing platform for the continuous screening of a spectrum of analytes ex vivo.

## Materials and methods

### Chemicals and materials

MBs (200 nm) were purchased from Ocean NanoTech (US). Tris(hydroxymethyl)aminomethane (Tris), hydrochloric acid, potassium chloride, magnesium chloride, sodium nitrite, potassium ferricyanide, lyophilized human serum, and lipopolysaccharide (LPS) were purchased from Sigma-Aldrich. Aqueous solutions were prepared using Milli-Q water. Recombinant human IFN-γ was purchased from the R&D Company. All DNA with the following sequences were synthesized and purified by Shanghai Sangon Biotechnology Co. Ltd. (Shanghai, China). According to the literature^24^, the selected hairpin aptamer probe sequence was 5′λ-Fc-GGG GTT GGT TGT GTT GGG TGT TGT GTC CAA CCCC-biotin-3′, where the underlined part indicates the sequence with affinity to IFN-γ. The compositions of the buffers used for the experiments are as follows: buffer for bead washing, 100 mM Tris–HCl, 1 mM EDTA, and 2 M NaCl pH 7.5 (buffer A); buffer for the immobilization of the primary aptamer onto the beads, 5 mM Tris–HCl, 0.5 mM EDTA, and 100 mM NaCl pH 7.5 (buffer B); detection buffer, Tris–HCl buffer (pH = 7.4) containing 100 mM Tris–HCl, 100 mM NaCl, 1 mM MgCl_2_, and 5 mM KCl (buffer C).

### Instrumentation

All electrochemical experiments were conducted using a CHI660E system (CH Instruments, Inc., Shanghai). The magnetic glassy carbon (GC) electrodes were 3 mm disks embedded in epoxy resin (Gaoss Union, China). All experiments utilized a Pt secondary electrode and a Ag/AgCl reference electrode. UV–vis absorption data were collected on a Shimadzu UV–vis spectrophotometer model 2450. DLS data were acquired using a Nano-ZS90 (Malvern) apparatus (Malvern Instruments, Malvern, UK).

### Preparation of MB-aptamer nanocomposites

MBs coated with streptavidin were washed with 500 μL of buffer A before use, as advised by the manufacturer. A suspension of 50 μL of nanobeads was introduced into a tube containing 50 μL of buffer A. After 15 min of incubation, the tube was positioned on a magnetic block to allow the precipitation of the nanobeads onto the bottom of the test tube; the supernatant was then removed, and the nanobeads were washed twice with 500 μL of buffer B and resuspended in 500 μL of buffer B. The nanobeads could also be prepared in advance and kept at 4 °C for several weeks before usage. To prepare the MB-aptamer nanocomposites, the aptamer solution (5 μM) was added to MB solution (100 ng mL^−1^) with a volume ratio of 1:2 and reacted for 2 h with shaking at room temperature. Then, the reaction tube was positioned on a magnetic block to allow the nanobeads to settle before removal of the supernatant and subsequently washed with buffer B twice. The MB-aptamer nanocomposites were obtained after repeating the washing steps twice and finally dispersed in buffer C for further usage. For the quantitation of aptamers loaded on MBs, 10 μL of the supernatant MBs was dropped onto the clean surface of a glassy carbon electrode, and then a cyclic voltammetry (CV) scan was performed in the buffer C solution from −0.2 to 0.6 V. The size distribution of the MB-aptamer nanocomposites was characterized by DLS in buffer C.

### Fabrication of microfluidic-based sensing devices

The customized microfluidic chip (Fig. 1) includes two layers: the bottom PDMS layer and the top PDMS layer. For the bottom PDMS layer with the microfluidic pattern, which was achieved by photolithography on 100 µm thickness Suλ on silicon wafers, the pattern includes the inlet sampling zone (1.2 mm in diameter), the sampling channel zone with a length of 25 mm × 0.3 mm, and the sensor loading zone (1.2 cm in diameter) connecting to the sampling outlet zone with a diameter of 0.8 mm. Afterwards, PDMS was poured directly on the pattern, which was left in an oven at 65 °C for 2 h for curing. Finally, the PDMS microfluidics chip was peeled off from the Su-8 mold for further use. For the top PDMS layer, three electrode sensors were fabricated on top of a single 525 µm thick crystal silicon wafer; over a native silicon dioxide (SiO_2_) insulated layer, 500 nm of gold was sputtered on top of a 20 nm chromium seed layer to provide defect-free adhesion of thin film gold electrodes as the working electrode (WE) and count electrode (CE), respectively^45^. Silver ink was deposited onto a 20 nm chromium seed layer to form the silver electrode as the reference electrode (RE)^46^. A silicon wafer with the sensing electrodes on top will be diced to suitable small pieces and will be put on the PDMS chip first, where the WE and CE are opposite to each other and RE is beside the WE. Liquid PDMS will be poured around each electrode sensor to form a uniform layer with exposure sensor surfaces. The electrode sensors were connected to the 3 separated electrostatic wires by conductive glue, which was connected to the external potentiostat. The final step is to bond the bottom PDMS pattern layer to the processed sensor/PDMS layer to form enclosed microchannels to ensure that the two PDMS layers match each other. A square magnet with a side length of 1.5 mm was mounted below the keyhole-shaped compartmentalized microchamber, as shown in Fig. 1. The image of the fabricated microfluidic device is illustrated in Fig. S1. All flow to the device was controlled via a syringe pump (PhD 2000, Harvard Apparatus). Sample solutions were continuously drawn into the device by engaging the waste pump at 16.7 μL/min.

### Electrochemistry measurement of IFN-γ

The MB-aptamer solution (100 μL) was flowed through the microfluidic device (Fig. 1) with a magnetic field underneath the sensor zone, and all MB-aptamers were eventually fixed in the sensor zone by the magnetic field. The oxidation signal of ferrocene was measured by using SWV in buffer C and scanning from −0.2 to 0.6 V. The real-time measurement was carried out by chronoamperometry in a series of different concentrations of IFN-γ by fixing the potential at 0.2 V for 5000 s.

### Procedure to measure the reversible capacity of the sensing interface

To study the regeneration of the MB-aptamer nanocomposite-based sensing interface, 1000 pg mL^−1^ IFN-γ was first flowed through the sensor zone to react with the MB-aptamer in the sensor zone to achieve the aptamer-IFN-γ conjugates. Then, the microfluidic chamber was washed with buffer B followed by a flow of buffer C for 2 h. Due to the reversible binding between the aptamer and IFN-γ, flow through with buffer C will induce dissociation of IFN-γ from the aptamer-IFN-γ conjugates to regenerate MB-aptamer nanocomposite-based sensing interface, which is available to bind IFN-γ again.

### Cell culture and measurement

Informed consent in this study was obtained under approved Human Research Ethics Committee protocols. Peripheral blood mononuclear cells (PBMCs) were purchased from Procell (Wuhan, China). They were cultured in a T25 cm^2^ flask containing the completed RPMI-1640 medium supplemented with 10% human serum AB heat inactivated, 10 U mL^−1^ penicillin, 1000 U mL^−1^ IL-2 and 1100 μg mL^−1^ streptomycin, 10 μg mL^−1^ gentamycin, 2 mM gentamine and 25 mM HEPES. The 6-well plate was used to tune the density cells to 1 × 10^6^/mL and incubated in a 37 ℃ 5% CO_2_ incubator to culture for a period of time. The cells were cultured to approximately 80–90% confluence before harvesting. During harvesting, the cells were washed twice with DPBS followed by trypsinization using 2 mL of trypsin to detach the cells from the flask. The trypsin was neutralized by adding 4 mL of fresh supplemented medium, and the harvested cells in the RPMI-1640 medium suspension were transferred into a centrifuge tube and centrifuged at 1500 rcf for 10 min. The supernatant was discarded, and the cells were resuspended in fresh medium. For preparation of IFN-γ samples, the cells with a density of 1 × 10^6^/mL were suspended in 1 mL of warm medium containing 0.1 μg mL^−1^ LPS to secrete IFN-γ for 0, 2, 4, 6, 8, and 20 h, respectively. Supernatants from these cells were collected in triplicate. The Nunc MaxiSorp 96-well plate and Galaxy plate reader were used for ELISA.

### Cytokine detection in human blood serum

Since under physiological conditions, cytokine concentrations are normally low or undetectable, we spiked IFN-γ into the serum samples^47^. Lyophilized serum was first reconstituted by dissolving 100 mg in 5 mL of deionized water with gentle stirring. Then, the serum was spiked with 50, 100, 150, and 200 pg mL^−1^ IFN-γ, and the recovery of a spiked sample was measured using the prepared sensing device.

## Supplementary information

Supplementary material

Editorial Summary

## References

[1] Sundberg TB, Xavier RJ, Schreiber SL, Shamji AF (2014). Small-molecule control of cytokine function: new opportunities for treating immune disorders. Curr. Opin. Chem. Biol..

[2] Mills KHG (2014). S-16: function and regulation of IL-17 cytokine family in infection and autoimmunity. Cytokine.

[3] Sumida K (2014). Abstract 3661: crucial roles of cytokine-signaling for alteration in functions of myeloid-derived suppressor cells. Cancer Res..

[4] Peng LS (2017). Tumor-associated monocytes/macrophages impair NK-cell function via TGFβ1 in human gastric cancer. Cancer Immunol. Res..

[5] Khalifeh MS, Stabel JR (2004). Effects of gamma interferon, interleukin-10, and transforming growth factor beta on the survival of *Mycobacterium avium* subsp. paratuberculosis in monocyte-derived macrophages from naturally infected cattle. Infect. Immun..

[6] Pandie S (2014). Diagnostic accuracy of quantitative PCR (Xpert MTB/RIF) for tuberculous pericarditis compared to adenosine deaminase and unstimulated interferon-γ in a high burden setting: a prospective study. BMC Med..

[7] Ullum H (1997). Low production of interferon gamma is related to disease progression in HIV infection: evidence from a cohort of 347 HIV-infected individuals. AIDS Res. Hum. Retroviruses.

[8] Tilg H (2002). Treatment of Crohn’s disease with recombinant human interleukin 10 induces the proinflammatory cytokine interferon γ. Gut.

[9] Hughes V (2013). Interferon gamma responses to proteome-determined specific recombinant proteins: potential as diagnostic markers for ovine Johne’s disease. Vet. Immunol. Immunopathol..

[10] Zaidi MR, Merlino G (2011). The two faces of interferon-gamma in cancer. Clin. Cancer Res..

[11] Zhao J, Chen C, Zhang L, Jiang J, Yu R (2012). An electrochemical aptasensor based on hybridization chain reaction with enzyme-signal amplification for interferon-gamma detection. Biosens. Bioelectron..

[12] Reece WH (2004). A CD4(+) T-cell immune response to a conserved epitope in the circumsporozoite protein correlates with protection from natural *Plasmodium falciparum* infection and disease. Nat. Med..

[13] Favre N, Bordmann G, Rudin W (1997). Comparison of cytokine measurements using ELISA, ELISPOT and semi-quantitative RT-PCR. J. Immunol. Methods.

[14] Liu G, Qi M, Zhang Y, Cao C, Goldys EM (2016). Nanocomposites of gold nanoparticles and graphene oxide towards an stable label-free electrochemical immunosensor for detection of cardiac marker troponin-I. Anal. Chim. Acta.

[15] Das TM (2013). Modelling vemurafenib resistance in melanoma reveals a strategy to forestall drug resistance. Nature.

[16] Qi M, Zhang Y, Cao C, Lu Y, Liu G (2016). Increased sensitivity of extracellular glucose monitoring based on AuNP decorated GO nanocomposites. RSC Adv..

[17] Wilson GS, Hu Y (2000). Enzyme-based biosensors for in vivo measurements. Chem. Rev..

[18] Vallée-Bélisle A, Plaxco KW (2010). Structure-switching biosensors: inspired by nature. Curr. Opin. Struct. Biol..

[19] Plaxco KW, Soh HT (2011). Switch-based biosensors: a new approach towards real-time, in vivo molecular detection. Trends Biotechnol..

[20] Nutiu R, Li Y (2003). Structure-switching signaling aptamers. J. Am. Chem. Soc..

[21] Cao C, Zhang F, Goldys EM, Liu G (2018). Advances in structure-switching aptasensing towards real time detection of cytokines. TrAC: Trends Anal. Chem..

[22] Kuswandi B, Huskens J, Verboom W (2007). Optical sensing systems for microfluidic devices: a review. Anal. Chim. Acta.

[23] Cohen N, Sabhachandani P, Golberg A, Konry T (2015). Approaching near real-time biosensing: microfluidic microsphere based biosensor for real-time analyte detection. Biosens. Bioelectron..

[24] Liu Y, Yan J, Howland MC, Kwa T, Revzin A (2011). Micropatterned aptasensors for continuous monitoring of cytokine release from human leukocytes. Anal. Chem..

[25] Syedmoradi L (2017). Point of care testing: the impact of nanotechnology. Biosens. Bioelectron..

[26] Centi S, Messina G, Tombelli S, Palchetti I, Mascini M (2008). Different approaches for the detection of thrombin by an electrochemical aptamer-based assay coupled to magnetic beads. Biosens. Bioelectron..

[27] Liu DM, Chen J, Shi YP (2018). Tyrosinase immobilization on aminated magnetic nanoparticles by physical adsorption combined with covalent crosslinking with improved catalytic activity, reusability and storage stability. Anal. Chim. Acta.

[28] Barthelmebs L, Hayat A, Limiadi AW, Marty JL, Noguer T (2011). Electrochemical DNA aptamer-based biosensor for OTA detection, using superparamagnetic nanoparticles. Sens. Actuators B Chem..

[29] Frohnmeyer E, Frisch F, Falke S, Betzel C, Fischer M (2018). Highly affine and selective aptamers against cholera toxin as capture elements in magnetic bead-based sandwich ELAA. J. Biotechnol..

[30] Erdem A, Duruksu G, Congur G, Karaoz E (2013). Genomagnetic assay for electrochemical detection of osteogenic differentiation in mesenchymal stem cells. Analyst.

[31] Zhang RQ, Hong SL, Wen CY, Pang DW, Zhang ZL (2017). Rapid detection and subtyping of multiple influenza viruses on a microfluidic chip integrated with controllable micro-magnetic field. Biosens. Bioelectron..

[32] Cheah JS, Yamada S (2017). A simple elution strategy for biotinylated proteins bound to streptavidin conjugated beads using excess biotin and heat. Biochem. Biophys. Res. Commun..

[33] Gosnell ME, Anwer AG, Cassano JC, Sue CM, Goldys EM (2016). Functional hyperspectral imaging captures subtle details of cell metabolism in olfactory neurosphere cells, disease-specific models of neurodegenerative disorders. Biochim. Biophys. Acta.

[34] Erdem, A., Congur, G. & Eksin, E. *Voltammetric aptasensor based on magnetic beads assay for detection of human activated protein C* (Springer, New York, 2016). 10.1007/978-1-4939-3197-2_13 26552824

[35] Hamner KL (2013). Using temperature-sensitive smart polymers to regulate DNA-mediated nanoassembly and encoded nanocarrier drug release. ACS Nano.

[36] Takahara M (2016). A salt-switchable artificial cellulase regulated by a DNA aptamer. Biomacromolecules.

[37] Idili A, Valléebélisle A, Ricci F (2014). Programmable pH-triggered DNA nanoswitches. J. Am. Chem. Soc..

[38] Delong JC, Murakami T, Yazaki PJ, Hoffman RM, Bouvet M (2017). Near-infrared-conjugated humanized anti-carcinoembryonic antigen antibody targets colon cancer in an orthotopic nude-mouse model. J. Surg. Res..

[39] Ranallo S, Prévost-Tremblay C, Idili A, Vallée-Bélisle A, Ricci F (2017). Antibody-powered nucleic acid release using a DNA-based nanomachine. Nat. Commun..

[40] Liu G, Qi M, Hutchinson MR, Yang G, Goldys EM (2016). Recent advances in cytokine detection by immunosensing. Biosens. Bioelectron..

[41] Zhou Q, Kwa T, Liu Y, Revzin A (2012). Cytokine biosensors: the future of infectious disease diagnosis?. Expert Rev. Anti Infect. Ther..

[42] Giljohann DA, Mirkin CA (2009). Drivers of biodiagnostic development. Nature.

[43] Song, C., Wang, Z.-G. & Ding B. Design, fabrication, and applications of DNA nanomachines. In: Fan, C. (ed.) *DNA Nanotechnology* (Springer, Berlin, Heidelberg, 2013).

[44] Liu Y, Matharu Z, Rahimian A, Revzin A (2015). Detecting multiple cell-secreted cytokines from the same aptamer-functionalized electrode. Biosens. Bioelectron..

[45] Zia AI (2015). Rapid and molecular selective electrochemical sensing of phthalates in aqueous solution. Biosens. Bioelectron..

[46] Walker SB, Lewis JA (2012). Reactive silver inks for patterning high-conductivity features at mild temperatures. J. Am. Chem. Soc..

[47] de Jager W, Bourcier K, Rijkers GT, Prakken BJ, Seyfert-Margolis V (2009). Prerequisites for cytokine measurements in clinical trials with multiplex immunoassays. BMC Immunol..

