# Supplementary material for: On-chip structure-switching aptamer-modified magnetic nanobeads for the continuous monitoring of interferon-gamma ex vivo
Source: Microsyst Nanoeng. 2019 Aug 26;5:35. doi: 10.1038/s41378-019-0074-1 (PMC6799845; doi:10.1038/s41378-019-0074-1)
Supplement: Supplementary file 1 — Supplementary material [file 41378_2019_74_MOESM1_ESM.docx]

**Supporting Information**

**On-Chip Structure-Switching Aptamer Modified Magnetic Nanobeads for Continuous Monitoring of** [**Interferon-gamma**](https://www.google.com/search?rlz=1C1GCEA_enAU753AU753&q=Interferon+gamma&spell=1&sa=X&ved=0ahUKEwiLwY_Gp9LZAhUIVbwKHd25AS4QkeECCCQoAA) **Ex Vivo**

*Guozhen Liu,^1,2^* Chaomin Cao,^2^ Shengnan Ni,^2^ Shilun Feng,^3^ Hui Wei^2^*

^1^Graduate School of Biomedical Engineering, ARC Centre of Excellence in Nanoscale Biophotonics (CNBP), Faculty of Engineering, The University of New South Wales, Sydney NSW 2052, Australia

^2^International Joint Research Center for Intelligent Biosensor Technology and Health, College of Chemistry, Central China Normal University, Wuhan 430079, P. R. China

^3^School of Engineering, Faculty of Science and Engineering, Macquarie University, Sydney, NSW 2109, Australia

*To whom correspondence should be addressed. Email: Guozhen Liu, [guozhen.liu@unsw.edu.au](mailto:guozhen.liu@unsw.edu.au)

**Fabrication of the microfluidic system**

The home-made microfluidic system consists of one PDMS chip and 3 integrated electrodes, and is illustrated in Figure S1.


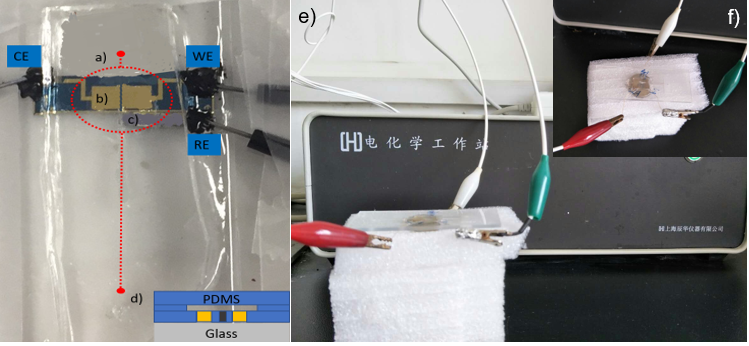


**Figure S1** The home-made microfluidic system: (a) pattern was drawn as red dash line; (b) The gold surfaces serve as the working electrode (WE) and counter electrode (CE), respectively with the dimension of 2.5 mm x 2.5 mm; (c) the silver wire works as the reference electrode (RE), with dimension of 1.2 mm x 10 mm; (d) the front view of the fabricated microfluidic system; (e) the microfluidic device connecting with the CHI potentiostat; (f) the top view of the chip in e).

**Optimization of parameters for preparation of MB-aptamer**

Figure S2 shows the SWV curves that 30 μL 5 μM aptamer solution reacted with magnetic beads solution in different volume for 2 h, respectively. After reaction, the tube was positioned on a magnetic block to allow the precipitation of the beads on the bottom of the test tube. After removing the supernatant, the beads were washed twice with buffer B, to obtain MB-aptamer complex. It was observed that the oxidation peak current of ferrocene increased with the volume of magnetic beads from 30 to 120 μL, and it reached a plateau when more than 60 μL of aptamer solution was added. Thus the optimised volume of magnetic beads solution was 60 μL which reacted with 30 μL 5 μM aptamer solution.


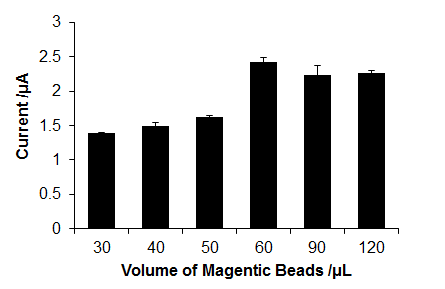


**Figure S2.** The relationship between the SWV current and the volume of 100 ng mL^-1^ magnetic beads solution reacted with 30 μL 5 μM aptamer solution.

**Optimization of the flow rate of the microfluidic device**

Different flow rate was applied to the microfluidic device to monitor the current response of the device to the 100 pg mL^-1^ IFN-γ (Figure S3). It was observed that 16.7 μL min^-1^ provided the maximum current suggesting the sufficient interaction between aptamers and the analyte IFN-γ.

**Figure S3.** The relationship between the flow rate and the current response of MB-aptamer based microfluidic device to 100 pg mL^-1^ IFN-γ.

**Regeneration of MB-aptamer based sensing interface**

The binding between aptamers and proteins was a reversible process, which was studied on the magnetic glassy carbon electrode. The electrochemical signal before and after stirring the IFN-γ bound MBs in buffer solution for 1 h (Figure S4) was recorded.


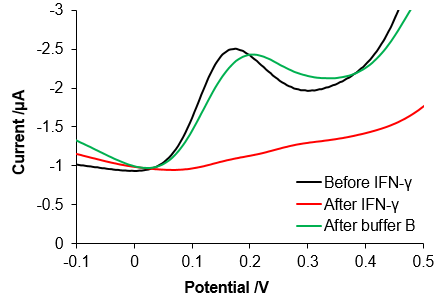


**Figure S4.** Reversible binding study of the MB-aptamer sensors by recording the SWV signal before and after stirring FN-γ bound surface in buffer B for 1 h.
