# Supplementary material for: On-chip structure-switching aptamer-modified magnetic nanobeads for the continuous monitoring of interferon-gamma ex vivo
Source: Microsyst Nanoeng. 2019 Aug 26;5:35. doi: 10.1038/s41378-019-0074-1 (PMC6799845; doi:10.1038/s41378-019-0074-1)
Supplement: Supplementary file 2 — Editorial Summary [file 41378_2019_74_MOESM2_ESM.docx]

# *Microsystems & Nanoengineering*

Sensors: High-sensitivity, real-time interferon gamma detector

Researchers have developed a device to continuously and precisely measure the concentration of the cytokine interferon gamma, a cellular signaling molecule which is an early indicator of many diseases, in real-time. Together with collaborators in Australia and China, Dr. Guozhen Liu of the University of New South Wales engineered a DNA hairpin that recognized interferon gamma and changed the configuration upon binding, which was attached to magnetic nanobeads on a microfluidic chip. The resulting device can detect interferon gamma at concentrations of just 6 pg per ml. Detection occurs because the DNA hairpin includes a ferrocene molecule which moves away from the sensor when the hairpin unfolds in response to interferon gamma, changing the electrochemical signal. This device provides a sensitive point-of-care sensing platform for continuous screening of interferon gamma levels, and future work could reconfigure it to detect different target molecules.

Related article manuscript number: MICRONANO-00763R

Article title: On-Chip Structure-Switching Aptamer Modified Magnetic Nanobeads for Continuous Monitoring of Interferon-gamma Ex Vivo

Corresponding author and affiliation/s: Guozhen Liu, University of New South Wales, Graduate School of Biomedical Engineering, Sydney, Australia

**About your Editorial Summary — please read**

**Before approving this Editorial Summary, please carefully check that (1) the summary text lists the correct author(s) and (2) the spelling and order of all author names and affiliations are correct.**

This **Editorial Summary** is based on your manuscript that was recently accepted for publication in *Microsystems & Nanoengineering*. It provides a non-specialist audience with a synopsis of your key research outcomes and conclusions. This value-added service provided by Springer Nature is designed to raise interest in your research across the broader community.

Springer Nature will publish the summary on the journal’s website, and it will be freely available under a under the CC BY licence (Creative Commons Attribution v4.0 International Licence) (see the journal website for details). We encourage you to re-use the summary to bring attention to your research; for example, you can host it on your own website and share it via social-networking platforms. Please attribute the summary to *Microsystems & Nanoengineering* and your article (e.g. by providing a link to your article) and do not make derivatives.

Please note that to maximise the usefulness of these summaries they must follow several stringent guidelines:
-- Spelling, punctuation and style are set according to *Nature* editorial guidelines. As this summary is aimed at non-expert readers, some concepts and technical terms will be simplified.
-- Total length must be no more than 135 words. It is likely that not all points in the paper will be covered.
-- The first sentence must be no more than 280 characters, including spaces, to allow use on microblogging sites.
-- The headline must consist of a brief generic subject identifier followed by a short description. No more than 10 words in total.

Please contact the editorial office ([mems_nano@mail.ie.ac.cn](mailto:mems_nano@mail.ie.ac.cn)) immediately with corrections should you find any factual errors in this Editorial Summary.
